# Genomic characterization, phylogenetic position and *in situ* localization of a novel putative mononegavirus in *Lepeophtheirus salmonis*

**DOI:** 10.1007/s00705-018-04119-3

**Published:** 2018-12-07

**Authors:** Arnfinn Lodden Økland, Are Nylund, Aina-Cathrine Øvergård, Renate Hvidsten Skoge, Heidi Kongshaug

**Affiliations:** 1 Fish Disease Research Group, Department of Biological Sciences, University of Bergen, Thormøhlensgt. 55, Pb. 7803, 5020 Bergen, Norway; 2 Sea Lice Research Centre, Department of Biological Sciences, University of Bergen, Thormøhlensgt. 55, Pb. 7803, 5020 Bergen, Norway

## Abstract

The complete genome sequence of a novel mononegavirus, Lepeophtheirus salmonis negative-stranded RNA virus 1 (LsNSRV-1), obtained from a salmonid ectoparasite, *Lepeophtheirus salmonis* was determined. The viral genome contains five open reading frames encoding three unknown proteins (ORF I, II and III), a putative glycoprotein (G), and a large (L) protein. Phylogenetic analysis placed LsNSRV-1 in the recently established mononegaviral family *Artoviridae.* LsNSRV-1 showed a prevalence of around 97% and was detected in all *L. salmonis* developmental stages. Viral genomic and antigenomic RNA was localized to nerve tissue, connective tissue, epithelial cells of the gut, subepidermal tissue, exocrine and cement glands, as well as the testis, vas deferens and spermatophore sac of male *L. salmonis* and the ovaries and oocytes of females. Viral RNA was detected in both the cytoplasm and the nucleoli of infected cells, and putative nuclear export and localization signals were found within the ORF I, III and L proteins, suggesting nuclear replication of LsNSRV-1. RNA interference (RNAi) was induced twice during development by the introduction of a double-stranded RNA fragment of ORF I, resulting in a transient knockdown of viral RNA. A large variation in the knockdown level was seen in adult males and off springs of knockdown animals, whereas the RNA level was more stable in adult females. Together with the localization of viral RNA within the male spermatophore and female oocytes and the amplification of viral RNA in developing embryos, this suggests that LsNSRV-1 is transmitted both maternally and paternally. Small amounts of viral RNA were detected at the site where chalimi were attached to the skin of Atlantic salmon (*Salmo salar*). However, as the RNAi-mediated treatment did not result in LsNSRV-1-negative offspring and the virus failed to replicate in the tested fish cell cultures, it is difficult to investigate the influence of secreted LsNSRV-1 on the salmon immune response.

## Introduction

The salmon louse (*Lepeophtheirus salmonis*), is a marine ectoparasite feeding on mucus, skin and blood of salmonids in the northern hemisphere [1, 2]. The salmon louse has a high reproductive capacity, and extensive farming of Atlantic salmon (*Salmo salar*) has led to an increase in host availability and density [2, 3]. Infestations of salmon lice are a serious problem for the salmon farming industry, with an estimated cost of €180 million each year [4]. The infestations have also been suggested to have a detrimental effect on wild salmonids [2, 3].

In the last few years, there has been a dramatic increase in the number of mononegaviruses discovered in arthropods, as new techniques for virus detection have been developed [5–12]. The order *Mononegavirales* consists of 11 families: *Rhabdoviridae*, *Filoviridae*, *Paramyxoviridae*, *Pneumoviridae*, *Bornaviridae*, *Nyamiviridae*, *Sunviridae*, *Mymonaviridae*, *Artoviridae, Lispiviridae*, and *Xinmoviridae* [13, 14]. The genomes of the mononegaviruses have the gene order 3’-UTR – core protein genes – envelope protein genes – RNA-dependent RNA polymerase gene – 5′-UTR [15]. For bornavirus genomes, this corresponds to the gene order 3′-UTR – nucleoprotein (N) gene – phosphoprotein (P) gene – matrix protein (M) gene – glycoprotein (G) gene – polymerase (L) gene – 5′-UTR [16–18]. Within the phosphoprotein gene, there is also an overlapping open reading frame (ORF) encoding the X protein, which is involved in regulation of polymerase activity [19, 20] and inhibition of type I interferon signalling and apoptosis [21, 22]. In the family *Nyamiviridae*, the genomes of the three viruses constituting the genus *Nyavirus* (Nyamanini virus, Midway virus and Sierra Nevada virus) exhibit the gene order 3′-UTR – N gene – ORF II gene – P gene – ORF IV gene – G gene – L gene – 5′-UTR. The ORF II protein of nyaviruses is a negative regulator of the polymerase activity, and ORF II and ORF IV are suggested to form a two-complex matrix protein [23]. The mymonaviruses are unique among the mononegaviruses because they encode the N protein in ORF II and have an ORF downstream of the L protein [24].

In 2014, Økland and colleagues described two rhabdoviruses infecting salmon lice: Lepeophtheirus salmonis rhabdovirus No 9 (LSRV-No9) and Lepeophtheirus salmonis rhabdovirus No 127 (LSRV-No127). These viruses are present in the glandular tissue of the louse and have a high prevalence in all developmental stages. Viral RNA is also present in the skin of the salmon surrounding the site where chalimi were attached, but the viruses do not replicate in selected fish cell cultures [9]. The viruses do not significantly affect the developmental rate, survival or fecundity of the salmon louse. However, infected lice appear to induce a dampened inflammatory response in salmon compared to virus-free lice [25]. Virus-free salmon louse strains have been established through RNAi-mediated treatment of the viruses, and studies have indicated that LSRV-No9 is transmitted both vertically and horizontally [26]. Recently, a related rhabdovirus genome was described from *Caligus rogercresseyi*: Caligus rogercresseyi rhabdovirus Ch-01 (CrRV-Ch01). CrRV-CH01 clusters phylogenetically with the two other caligid rhabdoviruses to form the newly created genus “*Caligrhavirus”* (awaiting ratification by the ICTV) within the family *Rhabdoviridae*. CrRV-Ch01 differs from LSRV-No9 and LSRV-No127 by having an additional ORF with unknown function [27]. Here, we describe the genome, phylogeny, tissue tropism and prevalence of a third putative virus from *L. salmonis*, Lepeophtheirus salmonis negative-stranded RNA virus 1 (LsNSRV-1), which shows similarities to artoviruses.

## Materials and methods

The complete description of the materials and the methods for Illumina sequencing and cell culturing systems has been reported elsewhere [9].

In short, a pooled sample of total RNA from five adult lice collected from different locations on the west coast of Norway was sequenced by BaseClear (BaseClear Group, The Netherlands) using Illumina next-generation sequencing. BF-2 (ATCCCCL91), ASK [28], CHSE-214 [29], and RT-Gill-W1 [30] cells were tested as possible culturing systems for the putative virus.

### Screening

A real-time RT-PCR assay (TaqMan probes) based on the putative L protein ORF of LsNSRV-1 was designed for relative quantification (Table 1). Assays targeting the elongation factor from salmon louse and the elongation factor alpha from Atlantic salmon were used as internal controls [31, 32]. A total of 157 *L. salmonis* from nine salmon farming sites in western Norway and from wild Atlantic salmon in the Oslofjord, 22 *C. rogercresseyi* from three salmon farming sites in Region X in Chile, and two *Caligus elongatus* from farmed Atlantic salmon in western Norway were tested for the presence of the LsNSRV-1 genome. To study the tropism of the virus, 16 *L. salmonis* were cut into five (male) or six (female) pieces: the anterior part of the cephalothorax, the middle part of the cephalothorax, the posterior part of the cephalothorax, the genital complex, the abdomen and the egg strings.

**Table 1:** Primers and probe for the TaqMan real-time RT-PCR assay targeting the L protein of LsNSRV-1 and primers used to make RNA probes for *in situ* hybridization targeting the ORF I protein

Code	Sequence	Position
Real time RT-PCR		
LsNSRV-1 L F	5′- CCG TTG CTT CCC CAT CAT T -3′	7376-7394
LsNSRV-1 L Probe	5′- AAT GAA ATT GTC TGG TCC TC -3′	7396-7415
LsNSRV-1 L R	3′- TCT GTG GAG ATT GAT GTA CAA ATT GTT -5′	7460-7434
*In situ* hydridization		
LsNSRV-1-ORFI F	5′- AGG GAA TTT CAA CAG TTA GGT TCT CA -3′	389-414
LsNSRV-1-ORFI R	3′- GGA AGG AAT ACC TCT GTA CCA TAC AGA -5′	1119-1093
RNA interference		
LsNSRV-1 -SYFw	5′- ATG CCT GTT CTT GAT ATT CCT ATC CTT GAC -3′	227-256
LsNSRV-1 -SYRev	3′- GTG TAC CAA TTC TCT CTG GAA GAG CAC GTG -5′	332-303

All samples were stored at – 20 °C. RNA was extracted using Tri Reagent^®^ (Sigma-Aldrich) according to the manufacturer’s protocol with a few modifications: the tissue was homogenized for seven minutes at 50 Hz using a Tissuelyser LT (QIAGEN) and a 5-mm bead, and an additional washing step with 1 ml 100% ethanol was included before air drying and elution with 50-100 µl of DEPC-treated water. An AgPath-ID™ One-Step RT-PCR Kit (Applied Biosystems™) and Applied Biosystems 7500 Real-Time PCR System (Applied Biosystems) were used for real time RT-PCR analysis with the following reactions: 1X RT-PCR buffer, 800 µM forward and reverse primer, 176 μM probe, 0.5 × RT-PCR enzyme mix, 2.0 μl template, and RNase-free water to a total volume of 12.5 μl. The reaction was run according to the standard protocol for the AgPath-ID™ One-Step RT-PCR kit (Applied Biosystems™).

### Determination of the 5′ and 3′ terminal sequences

RNA from infected lice was ligated to allow circularization and sequencing of the LsNSRV-1 genome termini. Total RNA was extracted from 5-7 lice using Tri Reagent (Sigma-Aldrich). To increase the efficiency of RNA ligation, the 5’ triphosphate residues of the RNA were removed by incubating 5 μg of total RNA with 5 units of 5’ RNA pyrophosphohydrolase (Rpph; New England Biolabs) in 40 μl of 1 × NEBuffer 2 for 30 min at 37 °C [33]. RNA cleanup was subsequently performed using an RNeasy Mini Kit (QIAGEN) according to the manufacturer’s recommendations. Purified dephosphorylated RNA (1 μg) was then ligated with 10 U of T4 RNA ligase (ThermoScientific) in 50 μl of 1 × reaction buffer for T4 RNA ligase supplemented with 0.1 mg of BSA per ml and 40 units of RNAseOUT (Invitrogen) for 1 h at 37 °C. For cDNA synthesis, 2.5 μl of ligated RNA was used directly as template for SuperScript III reverse transcriptase (SuperScript III First-Strand Synthesis System for RT-PCR, Invitrogen), with gene-specific primers annealing to the putative L gene in the genomic RNA. The cDNA was subjected to nested PCR with forward primers located within the 3′ end of the putative L gene and reverse primers located within the 5′ end of ORFI, using the Expand High Fidelity PCR system (Roche). Finally, the nested PCR products were gel purified (QIAquick Gel Extraction Kit, QIAGEN) and sequenced by the Sanger method using the same primers that were used for the nested PCR.

### *In situ* hybridization

*In situ* hybridization was performed on adult female and male lice according to Dalvin et al. [34] with modifications as described by Tröße et al. [35]. Digoxigenin-labelled (DIG-labelled) sense and antisense RNA probes were made for the ORF I gene using the primers listed in Table 1.

### Protein analysis

The theoretical isoelectric point (pI) and molecular mass (M_r_) of the putative proteins were calculated using ProtParam [36]. Phosphorylation and glycosylation sites were predicted using the NetPhos 3.1 server, the NetNGlyc 1.0 server and the NetOGlyc 4.0 server [37–39]. The Phobius web server was used to identify the signal peptide and transmembrane region of the G protein [40]. cNLS mapper was used to predict nuclear localization signals (NLS) [41], and LocNES [42], NESsential [43, 44] and NESmapper [45] were used to predict nuclear export signals (NESs). The COILS server [46] was used to predict coiled-coils domains. Protein sequences were aligned using MAFFT, and sequence identity, excluding gaps, was calculated using the identity distance algorithm in Unipro UGENE v1.26 [47].

### Phylogeny

Selected L protein amino acid sequences from members of the virus families *Nyamiviridae*, *Bornaviridae*, *Mymonaviridae*, *Artoviridae,* several unclassified negative-stranded RNA viruses related to members of these families, and at least one member of all mononegaviral genera approved by the ICTV were downloaded from the GenBank database. The 73 sequences were aligned using online MAFFT v7 [48], and poorly aligned regions were removed using trimAl [49], resulting in a sequence alignment of 565 amino acids. The best-fit model of protein evolution was determined by maximum-likelihood analysis using MEGA 6, based on the Bayesian information criterion (BIC). Phylogenetic trees were calculated using maximum-likelihood (ML) in MEGA 6 [50] with the LG + G + I + F model and 1000 bootstrap replications.

### Production of dsRNA and RNA interference

The 3′ end of ORF I was amplified by PCR using Q5 high-fidelity DNA polymerase (New England Biolabs) according to the supplier’s instructions, using the same T7 overhang primers as were used for the *in situ* hybridization (Table 1). The resulting PCR product (731 bp) was purified using a GenElute PCR Clean-Up Kit (Sigma-Aldrich), and double-stranded RNA (dsRNA) was synthesized using a MEGAscript^®^ RNAi Kit (Ambion) according to supplier’s instructions. RNA interference (RNAi) was performed as described previously by soaking of nauplii [51] or injection of pre-adults [52].

The dsRNA was further applied to a strain of LsRV-negative lice (LSOslo) [26] in an attempt to produce a strain that was free of all three viruses. The first approach was as described previously for the LsRVs [26], by immersion of nauplius larvae in 10 ng dsRNA per µl and a subsequent injection of pre-adult I females and pre-adult II males (600 ng/µl dsRNA) kept on fish at 12 °C. Samples for analysis were taken from copepodids from dsRNA-treated parents.

In the second approach, the concentration of dsRNA was increased. Nauplius I larvae from three pairs of egg strings were divided into four groups, where one group was treated with elution buffer while the other three groups were treated with 13, 20 and 27 ng of dsRNA per µl, respectively. Again, fish kept at 12 °C were infested with copepodids that were given 20 ng of dsRNA per µl at 7 days post-immersion (dpi), and 1000 ng of dsRNA per µl at the pre-adult I♀/II♂ stage (18 dpi). The lice were put back on the fish, and the egg strings were collected from the adult lice at 34 dpi and hatched in a single well flow-through system [53]. Samples for RNA isolation were taken from copepodids prior to infestation of fish, from pre-adult lice prior to injection, and from the adult lice and their offspring (hatched from their first egg string) at the copepodid stage. Pre-adult and adult lice were used individually for RNA isolation, while 20-40 copepodids hatched from the same egg string were pooled before RNA isolation.

## Results

### Genome

**Fig. 1:**
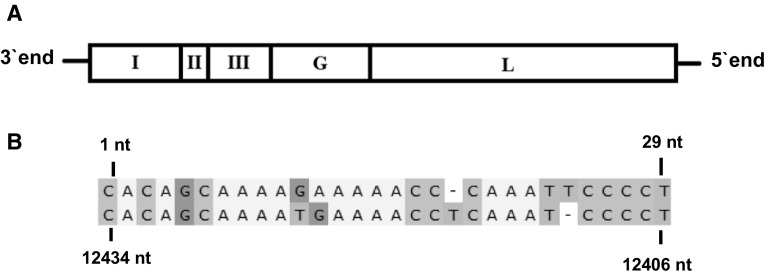
(**A**) Organization of the LsNSRV-1 genome with a schematic representation of coding regions. (**B**) The non-translated 3′-end and 5′-end regions of LsNSRV-1 exhibit inverse complementarity

A viral genome with sequence similarities to mononegaviruses was discovered in an Illumina sequence dataset from *L. salmonis*. The sequence was confirmed by Sanger sequencing, and the 5′- and 3′-terminal sequences were determined after circularization of the genome (GenBank accession number: MG489864). The complete 12,434-nucleotide (nt)-long negative-sense genome of the virus contains five ORFs with putative transcription initiation and termination sites in the order 3′-ORF I-ORF II-ORF III-G-L-5′. The G gene is in reading frame 1, while the ORF I, ORF II, ORF III and the L gene are in reading frame 3. The genome has a 173-nt-long 3′ leader region and a 129-nt-long 5′ trailer region. The first 29 nt of the 3′ leader region shows 86.7% reverse complementarity to the last 29 nt of the 5′ trailer region (Fig. 1).

### Protein genes

#### ORF I

The hypothetical ORF I gene is 2086 nt long and contains an ORF of 1986 nt encoding a putative protein of 661 amino acids (aa) (accession no: AUZ99695). The gene possesses a presumptive transcription initiation signal (TIS) (GAAACAA) and a transcription termination/polyadenylation signal (TTS) (TAAT(A)^5^). The protein has a molecular weight of 73.9 kDa and a pI of 6.5. A Blastp search of the putative ORF I protein found sequence similarity to hypothetical protein 1 of Běihǎi rhabdo-like virus 2 (YP009333446), hypothetical protein 1 of Běihǎi barnacle virus 8 (YP009333182), and a hypothetical protein of Pteromalus puparum negative-strand RNA virus 1 (PpNSRV-1) (APL97663). Aligning them to the ORF I protein sequence revealed 23%, 25% and 25%, amino acid sequence identity, respectively. Additionally, the ORF I protein shares similarities with an uncharacterized *Daphnia magna* protein (KZS21910) and an uncharacterized protein from *Dendroctonus ponderosae* (XP019755411), both with 24% amino acid sequence identity. LocNES, NESsential and NESmapper predicted the NESs _24_TMARALPERIGTLTL_38_ (score 0.513), _33_IGTLTL_38_ (score 0.65) and _26_ARALPERIGTLTLI_39_ (score 5.85), respectively. A proline-rich region (_634_PVVPAPAIRPPGPQLPPQNDGPPQDPNE_661_) was identified at the C-terminal end of the hypothetical protein, and a possible late domain was identified at amino acid position 221 (_221_YPDL_224_).

#### ORF II

The small putative ORF II gene is 337 nt long from its TIS (GAAATAA) to its TTS (TAACTT(A)^5^). The 5′-UTR of the ORF II gene overlaps the 3′-UTR of the ORF I gene by 33 nt. ORF II encodes a putative protein of 66 aa with a pI of 6.8 and a molecular weight of 7.6 kDa (accession no: AUZ99696). A Blastp search revealed no significant similarity to any known viral proteins. However, the ORF II protein shared slight sequence similarity with the condensation domains of two hypothetical proteins from the plant pathogenic fungi *Bipolaris victoriae* (XP_014554506) and *Bipolaris zeicola* (XP_007711178).

#### ORF III

The hypothetical ORF III gene encompasses 1327 nt from the putative TIS (GAAACAA) to the TTS (TAAG(A)^5^). The 1173-nt-long ORF III encodes a putative protein of 391 aa with a molecular weight of 42.9 kDa and a pI of 5.7 (accession no. AUZ99697). No similarity to other proteins was revealed using Blastp. c-NLS mapper predicted a bipartite NLS at amino acid position 15 (_15_KSGVKIIQTDVLDHLSESILEYDKKLKATKEP_46_) with a score of 7.7 [41]. Using the COILS server [46], two coiled-coil domains were predicted in the N-terminal and C-terminal end of the hypothetical protein. In all, 37 serine phosphorylation sites and 12 threonine phosphorylation sites were predicted by NetPhos 3.1. The C-terminus of the putative protein contains the two possible late domains, _360_PFSAP_364_ and _381_LDRLF_385_.

#### G gene

The putative glycoprotein (G) gene is 1860 nt long from the TIS (GAAATAA) to the TTS (TAAT(A)^5^). The 1794-nt-long ORF encodes a protein of 598 aa with a molecular weight of 66.9 kDa and a pI of 6.7 (accession no: AUZ99698). The putative G protein shows 24% amino acid sequence identity to a putative glycoprotein from PpNSRV-1 (APL97666) and a hypothetical protein from Húběi rhabdo-like virus 6 (APG78704). Additionally, a lower level of similarity was identified between the G protein and two uncharacterized arthropod proteins of similar size (KYN28643, XP015595137). A Phobius search of the G protein predicted a signal peptide and a transmembrane region at amino acid position 1-20 and 520-542, respectively. The putative glycoprotein contains eight predicted O-linked and three predicted N-linked glycosylation sites.

#### L gene

The putative L gene is 6556 nt in length, containing a 6534-nt-long ORF encoding a protein of 2178 aa (accession no. AUZ99699). The TIS and the TTS are assumed to be GAAACAA and TAAG(A)^5^, respectively. The putative L protein has a molecular weight of 247.5 kDa and a pI of 8.8. A Blastp search revealed sequence similarity between the putative LsNSRV-1 L protein and L proteins of the artoviruses PpNSRV-1 (APL97667, 30%), Běihǎi barnacle virus 8 (APG78659, 29%), Húběi rhabdo-like virus 6 (APG78705, 29%) and Húběi rhabdo-like virus 8 (APG78703, 28%), and Borna disease virus (NP042024, 26%), Nyamanini nyavirus (YP002905337, 25%), and *S. sclerotiorum* negative-stranded RNA virus 2 (ALD89145, 25%).

The L protein is the most conserved protein of the mononegaviruses, composed of six conserved blocks containing essential motifs for the structure and function of the polymerase [54]. Pairwise alignment of the L proteins from closely related viruses and selected members of the order *Mononegavirales* revealed that block III is the most conserved domain, while block VI is the least conserved (Table 2). In block II, the motif _504_KEREQK_509_ may be analogous to the motif KERELK in vesiculoviruses, which has been suggested to be involved in template recognition [54]. The subdomain III-C is the most conserved within block III, while subdomain III-D shows the least amino acid sequence similarity. A catalytic domain in the region from amino acid position 570 to 735 was detected using Motif scan [55]. A monopartite NLS was predicted at amino acid position 1495 (_1495_LVRIIKRWCKSY_1506_) by cNLS-mapper with a score of 7.5, and a possible bipartite NLS was detected by Motif scan at position 451 (_451_RKEWLLTPS-IKSDRR_466_). In block V, the sequence GSGT-72 aa-HR may correspond to the motif GSxT-(60–70 aa)-HR, which is essential for mRNA capping in nonsegmented negative-stranded RNA viruses [56].

**Table 2:** Percent amino acid sequence identity of LsNSRV-1 L protein domains and subdomains to related viruses and other members of the order *Mononegavirales*

Virus	L protein% identity	Blocks% identity						Subdomains block III (%)			
		I	II	III	IV	V	VI	III-A	III-B	III-C	III-D
PpNSRV-1(APL97667)	30%	34%	34%	38%	38%	32%	36%	69%	67%	90%	23%
Běihǎi barnacle virus 8 (APG78659)	29%	33%	34%	41%	30%	32%	31%	77%	52%	90%	46%
Běihǎi rhabdo-like virus 1(APG78668)	29%	34%	29%	42%	28%	29%	32%	54%	63%	90%	31%
Húběi rhabdo-like virus 5 (APG78806)	29%	30%	29%	36%	30%	34%	35%	69%	56%	70%	23%
Húběi rhabdo-like virus 6 (APG78705)	29%	29%	30%	40%	44%	33%	36%	62%	70%	90%	23%
Běihǎi rhabdo-like virus 2 (APG78672)	28%	29%	26%	43%	34%	29%	32%	69%	67%	100%	38%
Húběi rhabdo-like virus 8 (APG78703)	28%	30%	26%	33%	28%	27%	26%	54%	59%	60%	31%
Borna disease virus 1(NP 042024)	26%	15%	18%	24%	21%	21%	11%	38%	37%	50%	23%
Nyamanini nyavirus (YP002905337)	25%	17%	21%	27%	21%	19%	9%	38%	37%	70%	31%
*Sclerotinia sclerotiorum* negative-stranded RNA virus 2 (ALD89145)	25%	15%	19%	26%	21%	15%	6%	31%	52%	70%	31%
Avian metapneumovirus 15a(Q2Y2L8)	24%	13%	19%	21%	12%	16%	8%	23%	33%	40%	31%
Húběi rhabdo-like virus 7 (APG78729)	24%	18%	19%	24%	18%	15%	23%	38%	48%	50%	8%
Húběi rhadbo-like virus 4 (APG78632)	24%	16%	19%	24%	20%	17%	10%	38%	52%	60%	15%
Midway nyavirus (YP002905331)	24%	17%	22%	26%	20%	20%	11%	38%	37%	70%	31%
*Lepeophtheirus salmonis* rhabdovirus No127	24%	17%	18%	27%	21%	18%	16%	38%	41%	60%	23%
Newcastle disease virus B1(NP 071471)	23%	13%	15%	28%	15%	14%	28%	23%	44%	50%	31%
*Lepeophtheirus salmonis* rhabdovirus No9	23%	16%	17%	24%	13%	18%	19%	38%	44%	60%	31%
Vesicular stomatitis Indiana virus(NP 041716)	23%	16%	18%	23%	17%	17%	22%	38%	44%	50%	23%
Human orthopneumovirus (NP 056866)	22%	12%	20%	19%	15%	16%	5%	23%	37%	40%	23%
Zaire ebolavirus (NP 066251)	22%	17%	18%	24%	17%	17%	5%	38%	44%	40%	15%

#### Phylogeny

**Fig. 2:**
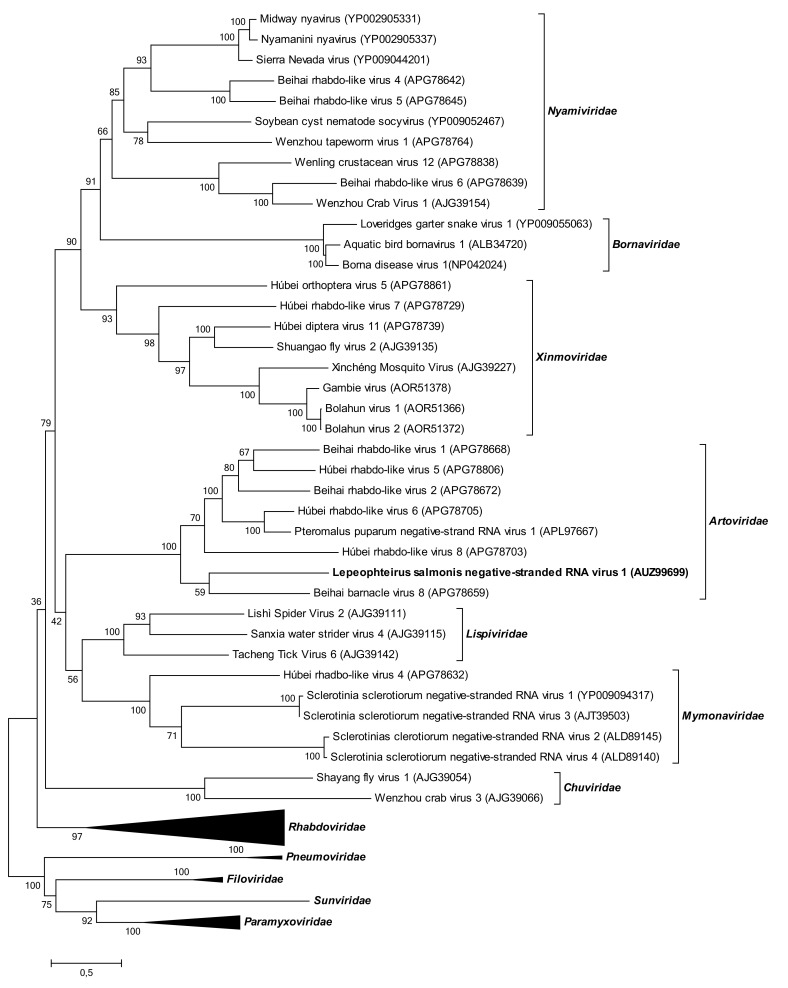
Phylogenetic position of LsNSRV-1 in relation to 73 other viruses from all genera of the 11 families of the order *Mononegavirales* and the family *Chuviridae* of the order *Jingchuvirales*. A maximum-likelihood tree based on alignment of 565 amino acids of the L-protein sequences is shown. The branch lengths reflect the evolutionary distance and are represented as the number of amino acid substitutions in proportion to the scale bar

The phylogeny inferred from comparison of L protein sequences of 73 mononegaviruses showed LsNSRV-1 clustering with members of the newly established mononegaviral family *Artoviridae* [14]. This family includes PpNSRV-1 (APL97667) and six novel arthropod viruses (Běihǎi barnacle virus 8 (APG78659), Běihǎi rhabdo-like virus 1 (APG78668), Húběi rhabdo-like virus 5 (APG78806), Húběi rhabdo-like virus 6 (APG78705), Běihǎi rhabdo-like virus 2 (APG78672) and Húběi rhabdo-like virus 8 (APG78703)) (Fig. 2). The *Artoviridae* clade has a bootstrap support value of 100, and clusters together with members of the families *Mymonaviridae* and the newly established family *Lispiviridae* with a bootstrap value of 42. The clade corresponding to the families *Artoviridae, Mymonaviridae and Lispiviridae* is separated from the remaining mononegaviral families in a larger clade with members of the families *Nyamiviridae*, *Bornaviridae* and *Xinmoviridae* with a support value of 79.

#### Prevalence and tissue association

Viral RNA from LsNSRV-1 is abundant in both male and female adult salmon lice and is detected in all stages of the louse life cycle, including developing embryos within egg sacs. The prevalence of the virus is approximately 97% (152 of 157). Tropism studies revealed that viral RNA is abundant throughout the body of the louse. Gill and kidney samples collected from six Atlantic salmon that were heavily infested with LsNSRV-1-positive salmon lice tested negative for the presence of viral RNA, while skin samples at the attachment site were weakly positive. Two *C. elongatus* samples tested positive for the presence of viral RNA by real-time RT-PCR. However, it is unknown if this is the same virus, as no attempts have been made to sequence LsNSRV-1 from *C. elongatus*.

#### *In situ* hybridization

**Fig. 3:**
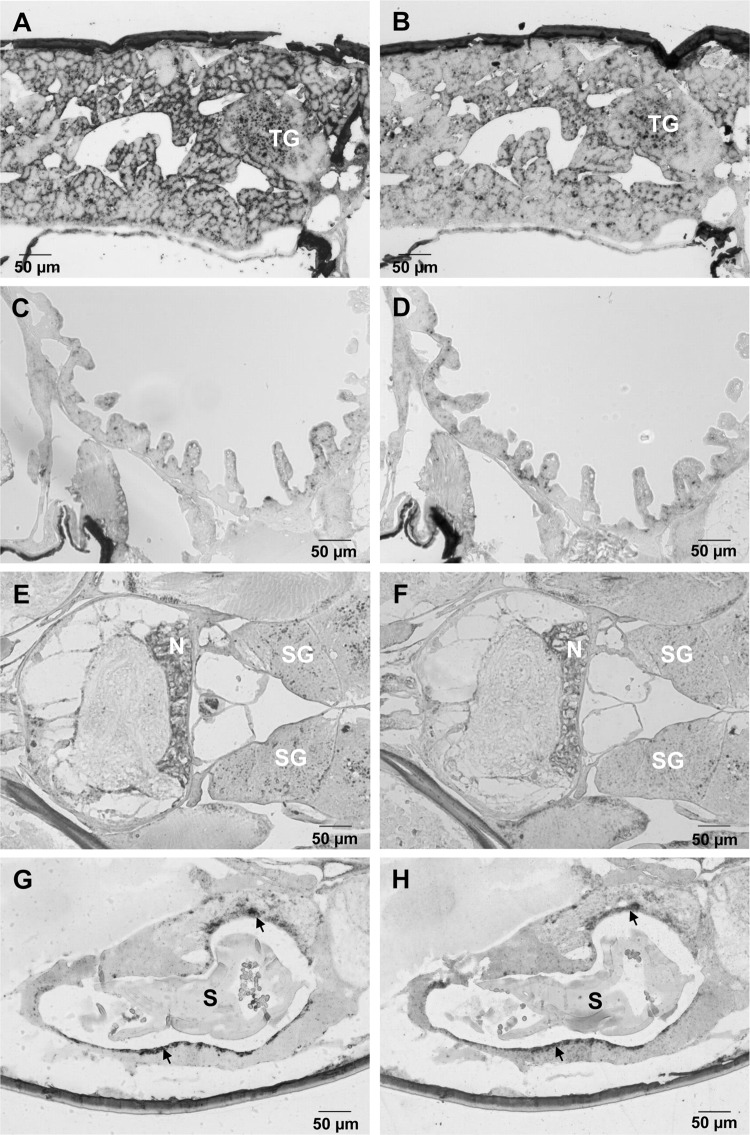
*In situ* hybridization targeting genomic and antigenomic RNA of LsNSRV-1. Heavy staining of antigenomic RNA is observed in sub-epidermal cells and in tegumental glands (TG) (**A**), while staining of genomic RNA is slightly weaker (**B**). The gut epithelial cells exhibit staining of both antigenomic (**C**) and genomic (**D**) RNA. The salivary glands (SG) show patches of antigenomic (**E**) and genomic (**F**) RNA staining, while nerve tissue (N) is heavily stained. The smaller cells of the spermatophore (S) are weakly stained for both antigenomic (**G**) and genomic (H) RNA, while the cells of the vas deferens (arrow) are heavily stained for both RNA strands

**Fig. 4:**
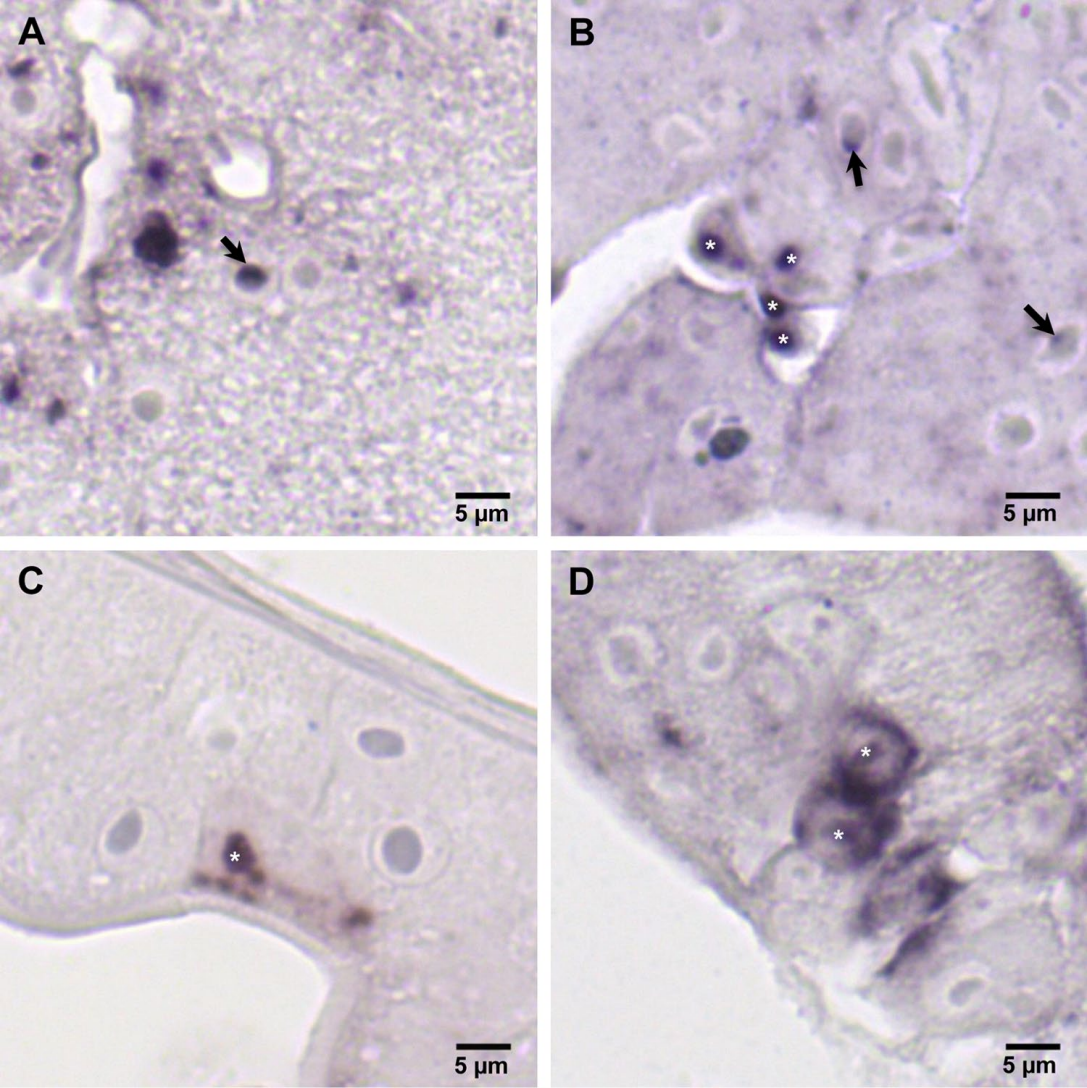
*In situ* hybridization reveals staining of the nucleolus and nucleus. Antigenomic staining of the nucleus (asterisk) and the nucleolus (arrow) is shown in a tegumental gland cell (**A**) and in subcuticular cells (**B**). Genomic staining of the nucleus is shown in an epithelial cell of the gut (**C**) and in subepidermal cells (**D**)

*In situ* hybridization revealed genomic RNA and expression of the ORF I gene in many L. salmonis tissues (Fig. 3). In the subepidermal tissue, denser staining of LsNSRV-1 RNA was observed as rings surrounding the nuclei of syncytia, particularly in the tissue facing the hemocoel. Dense patches were also observed in the salivary gland as well as in the tegumental type 1 and 2 glands. Weak staining and small dense patches were present in the cement gland, nerve tissue surrounding muscles, and epithelial cells of the gut. Weak and diffuse staining was seen in oocytes as well as in ovaries and testes. However, cells facing the lumen of the vas deferens and in the spermatophore sac were densely stained, and both genomic RNA and viral mRNA were localized to the small cells of the spermatophore wall. Genomic RNA and viral mRNA were detected in both the cytoplasm and the nucleus of affected cells (Fig. 4).

#### Cell culture

The virus failed to replicate in any of the cell cultures tested (BF-2, CHSE-214, ASK and RT-Gill-W1).

#### RNA interference

**Fig. 5:**
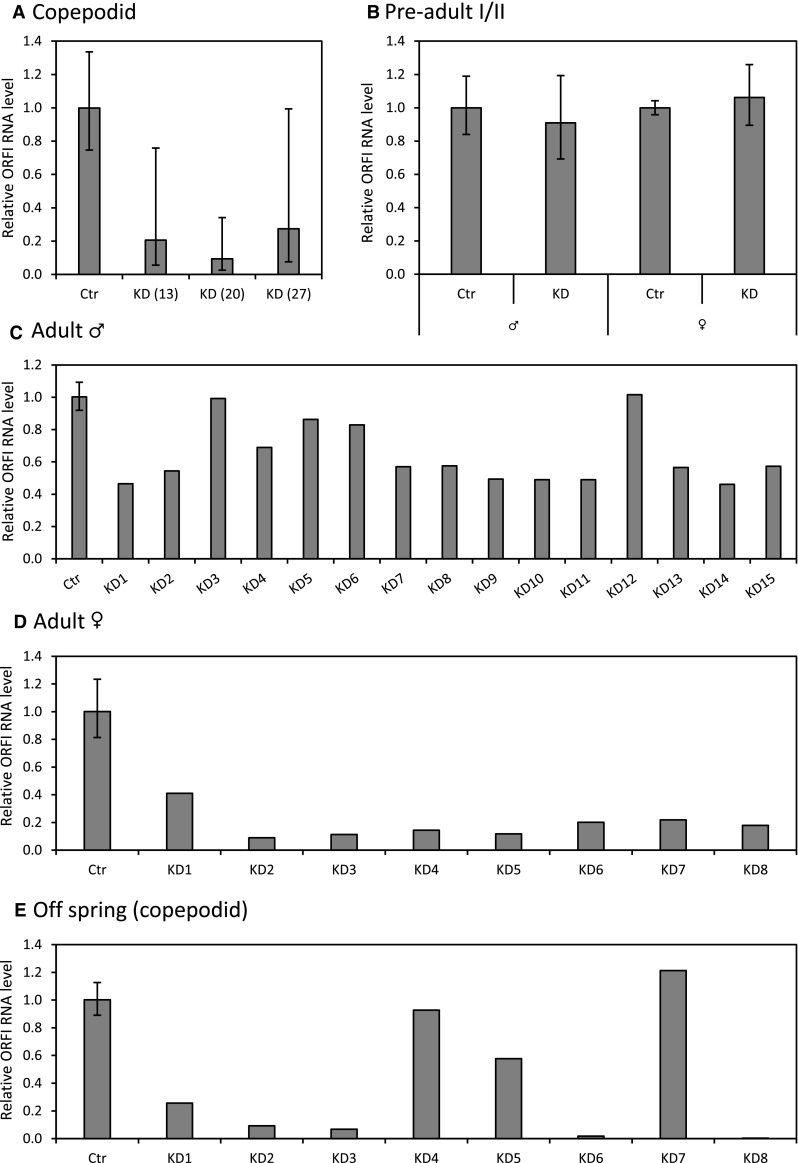
dsRNA treatment of LsNSRV-1-infected lice targeting ORF I. Viral RNA knockdown is shown as the relative RNA level (2^−ΔΔCt^) ± SD against control lice. **A**) Knockdown in copepodids (N = 3) 5 days post-immersion (dpi) in a 13, 20 or 27 ng/µl dsRNA solution at the nauplius I to II stage. **B** Knockdown in pre-adult I females and pre-adult II males at 18 dpi (N = 6). **C**-**D**) Knockdown in adult males and females at 34 dpi. The control is shown as an averaged knockdown (N = 6), while individual knockdown values are shown for the dsRNA-treated lice. **E**) Relative ORF I RNA level in the offspring from the knockdown females at the copepodid stage. The control is shown as the averaged knockdown (N = 3), while individual knockdown values are shown for each egg string

Opposite to what was previously shown for the LsRVs [25], the first RNA interference (RNAi) approach for LsNSRV-1 did not produce salmon louse offspring with decreased levels of viral RNA (results not shown). Therefore, a second round of RNAi was conducted in which higher concentrations of dsRNA were introduced into the lice. Of the three concentrations tested, maximum knockdown was achieved in copepodids treated with 20 ng of dsRNA per µl (Fig. 5A). These copepodids were allowed to infest fish, but at the pre-adult I/II stage, these lice had levels of viral RNA similar to those of the control group (Fig. 5B).

After a second round of RNAi by injection, the adult males had an average knockdown of 38.2% and the females had an average knockdown of 83.5% (Fig. 5C and D). While one female showed downregulation, only 59%, the remaining females showed a knockdown between 76 and 90% (Fig. 5D). The knockdown effect in the females did not correlate with the viral levels in their respective offspring, which varied between increased levels of viral RNA to a knockdown of up to 99% (Fig. 5E). However, large variation was also seen in the viral RNA levels in the males, with two individuals having the same amount of viral RNA as the controls while the others had a knockdown of 13.7 - 51%.

## Discussion

The family *Mononegavirales* currently consists of 11 families [14]. LsNSRV-1 clusters phylogenetically with members of the *Artoviridae*, a family containing seven other arthropod viruses, including PpNSRV-1. Artoviruses have five ORFs with gene lengths similar to those presented here for LsNSRV-1. ORF IV and ORF V share similarities with mononegaviral G proteins and L proteins, respectively. The possible functions of ORF I-III of artoviruses have not yet been examined.

The nucleoprotein of mononegaviruses is most commonly encoded by ORF I, with the exception of mymonaviruses where ORF I encodes a possible membrane protein and the nucleoprotein is encoded by ORF II [24]. The hypothetical protein encoded by LsNSRV-1 ORF I shares no characteristics with any known viral nucleoproteins, but it does show sequence similarity to several hypothetical ORF I proteins from unclassified mononegaviruses and PpNSRV-1. The LsNSRV-1 ORF I protein also contains a possible late domain, YPDL, corresponding to the YXXL late domain of membrane and Gag proteins of arenaviruses, paramyxoviruses and retroviruses [57–60]. Late domains are often proline-rich and are usually found in membrane proteins interacting with proteins of the endosomal sorting complexes required for transport (ESCRT) machinery, thus facilitating virion budding [61]. Such domains are also found in the nucleoproteins of arenaviruses, filoviruses, paramyxoviruses and retroviruses, where they are described to function as accessory factors for virion budding [61–64]. Whether the LsNSRV-1 ORF I protein primarily functions as a nucleoprotein or a membrane protein, the predicted late domain and the presence of an additional proline-rich region at the C-terminus suggest that the protein is involved in virion budding.

The phosphoprotein of mononegaviruses is a multifunctional protein acting as a cofactor for the RNA-dependent RNA polymerase complex [65]. For most mononegaviruses, the phosphoprotein is encoded by ORF II. ORF II may also encode other proteins in addition to the phosphoprotein [15, 19, 20, 66, 67]. For Nyamanini virus, the ORF II protein has been suggested to function as a matrix in a complex with the ORF IV protein [23]. The ORF II protein of LsNSRV-1 shows no resemblance to any of these proteins, and its putative function remains unknown. The ORF III of Nyamanini virus encodes an approximately 400-aa-long protein that functions as a polymerase cofactor [23]. The putative phosphoproteins of all three members of the genus *Nyavirus* are predicted to contain two coiled-coil regions at the N- and C-terminal ends of the protein [7]. The hypothetical ORFIII protein of LsNSRV-1 shows no sequence similarity to these proteins. Nevertheless, given that the LsNSRV-1 ORF III protein also has two predicted coiled-coil regions and is similar in size to the Nyamanini virus ORF III protein, combined with the fact that its gene is in the same position, it is likely that the LsNSRV-1 ORF III protein has a function similar to that of the putative phosphoproteins of nyaviruses. However, the domains _x_PFSAP_x_ and _x_LDRLF_x_ could represent the two late domains PT/SAP and LXXLF found in the matrix proteins of arenaviruses, filoviruses, rhabdoviruses and the Gag proteins of retroviruses [59, 68–70]. Thus, the LsNSRV-1 ORF III protein could also be a matrix protein involved in virion budding.

Based on sequence analysis of the hypothetical LsNSRV-1 ORF IV protein, its genome position, and the presence of a signal peptide and a transmembrane region, ORF IV is predicted to encode the G protein. The sequence similarity of the hypothetical LsNSRV-1 ORF V protein to other polymerases and the presence of several conserved domains related to the function of the polymerase indicate that ORF V encodes an RNA-dependent RNA polymerase.

Nucleorhabdoviruses and dichorhaviruses (family *Rhabdoviridae*), nyaviruses and bornaviruses replicate in the nucleus [23, 71, 72], and for bornaviruses, the nucleolus has been identified as the site of replication [73]. The nucleocytoplasmic trafficking of the ribonucleoprotein (RNP) complex is mediated by viral proteins possessing NLSs and NESs [74]. In bornaviruses, NLSs are found in the nucleoprotein, phosphoprotein, the non-structural protein p10, and the polymerase [75–78]. NLSs have also been reported to be present in the nucleoproteins and phosphoproteins of nucleorhabdoviruses and an unclassified *Culex tritaeniorhynchus* rhabdovirus [72, 79–81]. One leucine-rich domain in the nucleoprotein and one methionine-rich domain in the phosphoprotein of bornaviruses have been identified as NESs [82, 83]. Leucine-rich NESs have also been described in the C protein and nucleoprotein of morbilliviruses and the phosphoprotein of rabies virus [84–86]. Our analysis suggests the presence of NLSs in the ORF III protein and the polymerase, and one NES in the ORF I protein of LsNSRV-1, suggesting that of LsNSRV-1 replicates in the nucleus. There are examples of viruses with proteins exhibiting NLSs and NESs that replicate in the cytoplasm. The NLSs and NESs of both morbillivirus nucleoprotein and rabies virus phosphoprotein mediate nucleocytoplasmic trafficking of the protein, and both are involved in blocking of the IFN response [85–88]. However, the presence of both genomic RNA and viral mRNA of LsNSRV-1 in the nucleolus and the low efficiency of viral knockdown observed after treatment of lice with dsRNA targeting LsNSRV-1 ORFI indicate that this virus most likely replicates in the nucleus. While the cytoplasmic LsRVs have previously been shown to be entirely removed from lice by RNAi with only half the concentration of dsRNA used in this study [26], the presence of a nuclear reservoir of LsNSRV-1 might prevent efficient clearance of the virus by RNAi. Given that the virus particles have not been observed and that we were not able to cultivate the putative virus, one could argue that the virus is endogenous and that this prevents dsRNA-mediated removal of the virus. However, a viral genome incorporated into the *L. salmonis* genome with no exogenous phase should only be present in the cytoplasm as mRNA, and not as both mRNA and genomic RNA, as demonstrated by *in situ* hybridization. Moreover, the successful ligation and complementary termini of the putative viral genome strongly suggest that it is not incorporated in the host genome.

Arboviruses rely on horizontal transmission, mainly through feeding and infection of the arthropod’s vertebrate host [89, 90]. The dampened salmon immune response and higher parasitic success of lice infected with LSRV-No9 and LSRV-No127 suggest that these viruses have adapted to promote horizontal transmission. Like LSRVs, LsNSRV-1 is present in several glands that have ducts ending in cuticular pores on both the ventral and dorsal side of the salmon louse [91]. Viral RNA of LsNSRV-1 is also present in the gut and salivary glands. This could allow viral particles to be excreted and thus enable horizontal transmission. However, LsNSRV-1 has not been found in substantial amounts in the skin of salmon, and there is no evidence of replication in salmon. Vertical transmission of viruses in arthropods mainly relies on maternal transmission, though these viruses are also dependent on horizontal transmission in order to persist in the host population [89, 90, 92–94]. Vertical transmission from both males and females has currently only been reported in sigmaviruses, a possible reovirus, and PpNSRV-1. These viruses have been shown to persist in the host population without horizontal transmission [12, 95, 96]. Due to the presence of the viral genome of LsNSRV-1 in the genital products of both sexes of the salmon louse, and in the developing embryos and newly hatched nauplii, it is likely that LsNSRV-1 is transmitted vertically. The dense staining of LsNSRV-1 RNA in the vas deferens and spermatophore sac also indicate that the virus may be transmitted horizontally from males to females via seminal fluids as shown for LSRV-No9 [26]. Interestingly, a large variation in the amount of viral RNA was seen in the offspring of dsRNA-treated females, despite the relatively stable knockdown of viral RNA in adult females. Since a large variation in viral RNA levels was seen in the adult males as well, it is possible that LsNSRV-1 was transmitted vertically from the males to their offspring. Unfortunately, the experimental setup did not allow us to distinguish which male fertilized which female, and future production or identification of LsNSRV-1-free louse strains is needed to confirm such vertical transmission.

Understanding the role of the viruses infecting *L. salmonis* could be vital for the control of this parasite. Indeed, LSRV-No9 and LSRV-No127 infection enhances the parasitic success of *L. salmonis* [25]. LsNSRV-1 does not seem to infect salmon, as viral RNA was only present in the skin in small amounts, and it was not possible to cultivate the virus in the fish cell cultures that were tested. The close coexistence of salmon lice and salmon frequently exposes the viruses infecting salmon lice to potential new hosts. The host range of a virus is generally dependent on multiple genes encoding structural or non-structural proteins. Mutation, recombination or reassortment of these genes may facilitate a change in the host range of the virus [97]. Such events are probably very rare [98], and host shifts are most often observed between closely related hosts [99]. However, all arboviruses have undergone an interphyletic host shift at some point in time, and it has also been shown by Li et al. [100] that the plant pathogen tobacco ringspot virus underwent an interkingdom host shift to be able to infect and replicate in the honey bee *Apis mellifera*. It is therefore possible that viruses in the blood-feeding salmon lice could pose a risk to Atlantic salmon. Surveying and characterization of the virome of salmon lice could thus be of value for the fish farming industry. Clearly, more research is needed to clarify the effect of LsNSRV-1 on its host and to assess the risk of a host shift to Atlantic salmon.
